# Analysis of the genes controlling three quantitative traits in three diverse plant species reveals the molecular basis of quantitative traits

**DOI:** 10.1038/s41598-020-66271-8

**Published:** 2020-06-22

**Authors:** Meiping Zhang, Yun-Hua Liu, Wenwei Xu, C. Wayne Smith, Seth C. Murray, Hong-Bin Zhang

**Affiliations:** 1 College of Life Science, Jilin Agricultural University, Changchun, Jilin, 130118 China; 2 Department of Soil and Crop Sciences, Texas A&M University, College Station, Texas 77843 United States of America; 3 Texas A&M AgriLife Research, Lubbock, Texas 79403 United States of America

**Keywords:** Agricultural genetics, Plant genetics, Quantitative trait

## Abstract

Most traits of agricultural importance are quantitative traits controlled by numerous genes. However, it remains unclear about the molecular mechanisms underpinning quantitative traits. Here, we report the molecular characteristics of the genes controlling three quantitative traits randomly selected from three diverse plant species, including ginsenoside biosynthesis in ginseng (*Panax ginseng* C.A. Meyer), fiber length in cotton (*Gossypium hirsutum* L. and *G. barbadense* L.) and grain yield in maize (*Zea mays* L.). We found that a vast majority of the genes controlling a quantitative trait were significantly more likely spliced into multiple transcripts while they expressed. Nevertheless, only one to four, but not all, of the transcripts spliced from each of the genes were significantly correlated with the phenotype of the trait. The genes controlling a quantitative trait were multiple times more likely to form a co-expression network than other genes expressed in an organ. The network varied substantially among genotypes of a species and was associated with their phenotypes. These findings indicate that the genes controlling a quantitative trait are more likely pleiotropic and functionally correlated, thus providing new insights into the molecular basis underpinning quantitative traits and knowledge necessary to develop technologies for efficient manipulation of quantitative traits.

## Introduction

Most traits or biological processes that are important to agriculture and human health and medicine are quantitative traits or complex traits. These traits include, but are not limited to, crop yield, crop quality and plant response to biotic and abiotic stresses for crop plant species; milk, meat or egg productivity for livestock species; and height, weight, obesity, diabetes and cancers for humans. Classical quantitative genetics defines that quantitative traits are traits that can be phenotyped only quantitatively, vary continuously and present in a normal distribution. This phenomenon has been explained as a quantitative trait is controlled by numerous genes, with each gene having relatively small effect, and readily affected by environments. Therefore, quantitative traits are also known as polygenic traits.

Because of their importance in agriculture and human health and medicine, quantitative traits have been extensively studied through classical genetics up to the latest genome technologies, such as quantitative trait locus (QTL) mapping and genome-wide association study (GWAS). These studies have consistently confirmed the known of quantitative genetics that a quantitative trait is controlled by numerous genes, but showed that some of the genes controlling a quantitative trait have major effects and others have minor effects. Based on the results of QTL mapping and GWAS, from several to dozens of the genes controlling a quantitative trait of biological or economic importance have been cloned, annotated and characterized individually in the past 30 years. These studies showed that a quantitative trait is controlled by genes with diverse biochemical functions (for references, see below). However, many questions remain unknown about the molecular basis underpinning a quantitative trait. These questions include, but are not limited to, what kind of genes control quantitative traits; if a quantitative trait or a biological process is controlled by numerous genes, whether there is any relationship among the multiple genes; and if there is a relationship, how they are related to shape the performance of the trait. The shortage of such knowledge is limiting our understanding of the molecular mechanisms underpinning a quantitative trait and also development of new or advanced technologies, such as gene editing, for enhanced breeding and for enhanced medical profession.

In the present study, we address these questions using the cloned genes controlling three quantitative traits that were randomly selected from three diverse plant species. These three quantitative traits included ginsenoside biosynthesis in ginseng, *Panax ginseng* C.A. Meyer; fiber length (upper-half mean length, UHML) in cotton, *Gossypium hirsutum* L. and *G. babardense* L., and grain yield in maize, *Zea mays* L. Ginseng is one of the most important medicinal herbs in Asia and North America, cotton is the world’s leading natural fiber crop supporting the world’s textile industry, and maize is the world’s leading crop for food, feed and biofuel. These three quantitative traits are all economically crucial to agriculture and related industries. Importantly, use of the three quantitative traits in three diverse species allowed identification of molecular characteristics of quantitative traits that are common and applicable across quantitative traits and across species. Furthermore, use of the three species that were grown across three diverse environments would minimize the effect of environmental variation on the findings of the molecular basis underpinning a quantitative trait. Therefore, the findings of this study are also applicable across environments.

Gene expression, including RNA alternative splicing and transcript activity, is essential for a gene to function for or to control the development and phenotype of a trait. Therefore, analysis of gene expression has been widely used for molecular analysis of quantitative traits^1^, in combination with the linkage disequilibrium-based quantitative trait research, such as QTL mapping, GWAS and gene expression QTL (eQTL) mapping^2–8^, to identify candidate genes controlling a quantitative trait. Therefore, in this study we especially investigated the expression activities of the genes controlling these three quantitative traits. The findings of this study provide not only new insights into the molecular mechanism underpinning a quantitative trait, but also a molecular basis necessary to design new or advanced and efficient technologies, such as gene editing, for enhanced breeding in crops and livestock, and for enhanced medicine in humans. Finally, the findings of this study are also useful for genome-wide identification of the genes controlling a quantitative trait.

## Materials and Methods

### Plant materials

Three diverse plant species, ginseng, cotton and maize, with one population for each species, were used in this study. Maize is a monocotyledonous paleopolyploid species whereas ginseng and cotton are dicotyledonous allotetraploid species. Maize and cotton are annual and ginseng is perennial. Therefore, these three species have a wide representation for economic plant species. The ginseng population consisted of 42 cultivars and germplasm lines of a ginseng GWAS panel. The cotton population consisted of 198 recombinant inbred lines (RILs) and their two parents, *G. hirsutum* acc. TAM 94L-25 and *G. barbadense* acc. NMSI 1331. The maize population consisted of 89 intermated recombinant inbred lines (IRILs) and their inbred parents, B73 and Mo17. The RILs of the cotton population at F_10_ generation were used for this study. The maize population was a part of the intermated B73 x Mo17 (IBM)−302 population that is widely used for maize genome research^9^. The maize population has been grown and self-pollinated for many generations in Texas, USA. The IRILs of the maize population at F_14_ generation were used for this study.

### Field trials

Considering that various environments might influence the findings of this study on the questions whether and how the genes controlling a quantitative trait are related and whether their relationship, if any, contributes to the performance of the trait, due to G x E interaction, we planted the three populations in three diverse environments. The ginseng population was grown at Jingyu, Jilin, China (42°06′N 126°30′E) located in the Temperate Climate Zone from 2009 to 2013. The field trial was displayed in a randomized complete block design (RCBD) with two replicates (Supplemental Fig. S1A). Each plot had a single row of 40 plants spaced by 30 cm, with a distance of 100 cm between rows. The field practice, such as fertilization, weed and disease control, and irrigation, followed those locally used for ginseng production.

The cotton population was grown at College Station, Texas, USA (30°36′N 96°18′W) located in the Humid Subtropical Climate Zone in 2011. The field trial was plotted in RCBD with three replicates (Supplemental Fig. S1E). Each plot consisted of a single row with a length of approximately 183 cm (6 feet) and a distance of approximately 102 cm (40 inches) between rows. Five plants were planted in each plot guarded by one red cotton plant at each plot end, with a distance of approximately 30 cm (1 foot) between plants. To control the effects of field microenvironments on the phenotypes of the population, the parents of the population were used as checks and distributed in the trial, with one parent entry in every 20 entries. The field trial was guarded by two rows of the red cotton plants. The field practice followed those locally used for standard cotton field trials and production.

The maize population was grown at Halfway, Texas, USA (33°34′N 101°53′W) located in the Semiarid Steppe Climate Zone and College Station, Texas in 2010. The field trial was performed in RCBD with two replicates (Supplemental Fig. S11). Each plot was 610 cm (20 feet) long and 152 cm (5 feet) wide, consisting of two rows. Forty seeds were planted in each row and at the seedling stage, the number of plants per row were thinned into 35 plants per row. Therefore, each plot contained 70 plants, making 33,500 plants per acre. The field trial was guarded with two rows of a maize purple-colored cultivar. The field practice followed those locally used for standard maize yield trials and production.

### Target traits and phenotyping

Three quantitative traits were used for this study, including ginseng ginsenoside biosynthesis, cotton fiber length and maize grain yield. These three traits represent three typical quantitative traits showing normal distribution in plants, as described in quantitative genetics (see Supplemental Fig. S1) and have been extensively studied in our laboratories. For the ginseng population, the total ginsenoside contents of four-year-old plant roots (Supplemental Fig. S1C), presented in mg/g (Supplemental Fig. S1D), were obtained from Zhao *et al*.^10^.

For the cotton population, fiber length, presented as Upper Half Mean Length (UHML) (Supplemental Fig. S1G), was measured from all three replicates of the field trial. When the fibers were matured, all fiber bolls were harvested from the entire plot and ginned in the Cotton Improvement Laboratory, Texas A&M AgriLife Research, College Station, Texas, USA. A sample of the fibers from each replicate of each line was used to measure UHML using High-Volume Instrumentation (HVI) at the Fiber and Biopolymer Research Institute, Texas Tech University, Lubbock, Texas, USA. The fiber length of each line was the mean fiber length of its three replicates (Supplemental Fig. S1H) because the fiber length was highly reproducible between replicates.

For the maize population, grain yield (Supplemental Fig. S1K), presented in grain weight (g) per plant, was measured from both replicates of the field trial. When the ears ripened, the number of plants and ears were counted per plot, 10 plants were randomly selected from the middle of each plot, with 5 plants per row, and the ears of the selected plants were harvested. The ears were naturally dried, and grains were threshed and weighed. The grain yield of each line was the mean grain yield of the 20 plants sampled from its two replicates (Supplemental Fig. S1L). The variation distribution of each of the three traits (Supplemental Fig. S1D,H,L) confirmed that they are all typical quantitative traits showing normal distributions.

### Published genes

The genes that were previously cloned from different genetic sources by different researchers and that were shown to control ginseng total ginsenoside content or ginsenoside biosynthesis, cotton fiber length or maize grain yield were used in this study. To have the genes controlling these three traits, an extensive literature search was conducted as of December 2014. In addition, we also searched for the published genes that control trichrome in the plant model species, Arabidopsis [*Arabidopsis thaliana* (L.) Heynh.], because cotton fiber is developed from trichome^11^, and the published genes that control grain yield in the monocot plant model species, rice (*Oryza sativa* L.), because some rice grain yield genes were shown to have equivalent biological functions in maize^12–15^. Therefore, the Arabidopsis trichrome genes as the orthologues of cotton fiber length genes were used with the cotton fiber length genes, and the rice grain yield genes as the orthologues of maize grain yield genes were used with the maize grain yield genes in this study.

The homologues of the ginseng ginsenoside biosynthesis genes were identified by BLAST from the transcriptome of a four-year-old ginseng plant^16^ using the sequences of the published genes as queries, with query cover ≥300 bp, identity ≥90%, and *E*-value ≤ 1.0E-06. The homologues of the cotton fiber length genes and the cotton orthologues of the Arabidopsis trichrome genes were identified from the 10-dpa (days post-anthesis) developing fiber transcriptome of the cotton population’s parent, TAM 94L-25 (ref. ^17^), using the sequences of the published genes as queries, with query cover ≥300 bp, identity ≥90%, and *E*-value ≤ 1.0E-06. The homologues of the maize grain yield genes and the maize orthologues of the rice grain yield genes were identified from the 13-leaf developing top ear shoot transcriptome of the maize population’s parent, B73 (ref. ^17^), using the sequences of the published genes as queries, with query cover ≥300 bp, identity ≥90%, and *E*-value ≤ 1.0E-06. The sequence identities lower than 100% were used for identification of the homologous or orthologous genes controlling the traits from the databases because genetic diversity may exist between the sources from which the genes were cloned and those of the databases. To validate the BLAST search results and further characterize the homologues or orthologues of the cloned genes, they were annotated and subjected to gene ontology (GO) analysis using Blast2GO^18^. The annotation confirmed the BLAST search results.

### Gene expression profiling

The expressions of the ginsenoside biosynthesis gene transcripts in four-year-old ginseng plant roots (Supplemental Fig. S1B) were extracted from a transcriptome database of four-year-old plant roots of the ginseng GWAS panel generated by shotgun RNA-seq.^19^. Supplemental Fig. S2A shows the expression variation of the gene transcripts among the germplasm lines of the ginseng GWAS panel. The expressions of the fiber length gene transcripts and those of the cotton orthologues of the Arabidopsis trichrome genes in cotton 10-dpa developing fibers (Supplemental Fig. S1F) were extracted from a transcriptome database of the 10-dpa developing fibers of the cotton population generated by shotgun RNA-seq.^17^. Supplemental Fig. S2B shows the expression variation of the gene transcripts among the RILs of the cotton population. The expressions of the maize grain yield gene transcripts and those of the maize orthologues of the rice grain yield genes in maize 13-leaf developing top ears (Supplemental Fig. S1J) were extracted from a transcriptome database of the maize 13-leaf developing top ears of the maize population generated by shotgun RNA-seq.^17^. Supplemental Fig. S2C and D show the expression variation of the gene transcripts among the lines of the maize population. Zhang *et al*.^17^ showed that the expressions of individual transcripts quantified by the shotgun RNA-seq method were highly reproducible, with a Spearman’s correlation coefficient varying from *r* = 0.90 to *r* = 0.98 (*P* = 0.00E + 00), between plants (biological replicates) grown within the same field trial replicate and different field trial replicates. Shot-gun or full-length RNA-seq was the method of choice that was the most proper to quantify the expressions of genes and their individual transcripts, while other methods, including real-time quantitative PCR, could not properly quantify the expressions of individual transcripts and genes^17^.

### Gene co-expression network construction

The co-expression networks of the genes were constructed using the BioLayout *Expression*^3D^ software^20^.

### Statistical analysis

The analysis of variance (ANOVA), followed by least significant difference (LSD), Student’s *t*-test and Chi-square test were performed using Excel, the customized R scripts (R 3.0.1) or the statistical package IBM SPSS Statistics 22. The two-tailed significance level was applied for all statistical analyses.

## Results

### Molecular characteristics of the genes controlling quantitative traits

**Figure 1:**
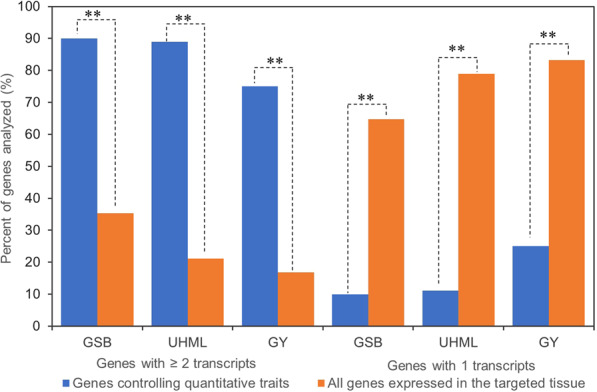
Up- and down-enrichment of the genes controlling a quantitative trait that are subjected to RNA alternative splicing. Of the genes controlling ginseng ginsenoside biosynthesis (GSB), cotton fiber length (UHML), maize grain yield (GY), those spliced into two or more transcripts were up-enriched, while those spliced into single transcripts were down-enriched, relative to those of all genes expressed in the tissue. The asterisks “**” indicate the significance of *P* ≤ 0.01 determined by Chi-square test for which the percentages of the genes out of all genes expressed in the tissue with two or more transcripts and the genes with single transcripts were used to calculate the expected ones (see ref. ^17^).

Literature search found 10 genes that were shown to be involved in ginsenoside biosynthesis in ginseng (Supplemental Table S1)^23^–^34^, 18 genes controlling cotton fiber length and two genes controlling Arabidopsis trichrome (Supplemental Table S2)^23^–^34^ and eight genes controlling maize grain yield and 18 genes controlling rice grain yield (Supplemental Table S3)^13^–^15^,^54^–^77^. These genes were cloned totally independently by different researchers from different genetic sources using different gene cloning methods, including map-based cloning, T-DNA or transposon insertional mutagenesis, RNA interference (RNAi), antisense gene expression repression, gene overexpression, and/or gene regulation analysis. Sequence analysis showed that the genes controlling the same quantitative trait are different in nucleotide sequence and have very different annotations (Supplemental Tables S1–S3). BLASTing the transcriptome of a four-year-old ginseng plant^10,16^ using the sequences of the ten ginseng ginsenoside biosynthesis genes as queries revealed that nine (90%) of the 10 genes were alternatively spliced into multiple transcripts and one had a single transcript, with a range of 1–19 transcripts and an average of 6.7 transcripts per gene (Supplemental Table S4). BLASTing the transcriptome of cotton developing fibers^17^ using the sequences of the 18 cotton fiber length genes and two Arabidopsis trichrome genes as queries identified that 17 (85%) of the 20 genes were alternatively spliced into multiple transcripts and three each had a single transcript, with a range of 1–69 transcripts and an average of 14.8 transcripts per gene (Supplemental Table S5). BLASTing the transcriptome of maize developing ear shoots^17^ using the sequences of 8 maize grain yield genes and 18 rice grain yield genes as queries identified that six (75%) of the eight maize grain yield genes were alternatively spliced into multiple transcripts and two each had a single transcript, with a range of 1–13 transcripts and an average of 2.4 transcripts per gene (Supplemental Table S6). Fourteen (78%) of the maize orthologues of the 18 rice grain yield genes were alternatively spliced into multiple transcripts and four each had a single transcript, with a range of 1–32 transcripts and an average of 5.3 transcripts per gene. These results together indicated that 75% or more of the genes controlling a quantitative trait, ginseng ginsenoside biosynthesis, cotton fiber length or maize grain yield, are pleiotropic, having multiple functions, if each of their transcripts has a different function. Chi-square test showed that the numbers of the genes that were alternatively spliced into multiple transcripts were significantly enriched (*P* ≤ 0.01), relative to the number of transcripts per gene for all genes expressed in these tissues^17^ (Fig. 1). Moreover, we categorized the transcripts of the genes or orthologues controlling ginseng ginsenoside biosynthesis, cotton fiber length, and maize grain yield, respectively, using gene ontology (GO). The results showed that the transcripts of the genes controlling these traits were categorized into 4 (Supplemental Fig. S3A), 26 (Supplemental Fig. S3B), and 23 (Supplemental Fig. S3C,D) GO subcategories at Level 2, respectively, suggesting that the genes controlling a quantitative trait have very diverged biochemical functions.

### Correlation of the expressions of genes controlling a quantitative trait with the phenotype of the trait

Since the vast majority of the genes controlling the three quantitative traits were alternatively spliced into multiple transcripts, the question was whether all the transcripts spliced from each of the genes contributed to the phenotype of the targeted trait. To answer this question, we conducted correlation analysis between expression variations of individual gene transcripts (Supplemental Fig. S2) and phenotype variation of the three traits (Supplemental Fig. S1D,H,L), respectively, in a GWAS panel (ginseng) or a bi-parental population (cotton and maize). We hypothesized that if the expression of a gene transcript was correlated with the phenotype variation of a trait, the transcript was considered to be likely involved in the phenotype development of the targeted trait. This is because expression is essential for a gene to function and contribute to the performance of a trait. The results showed that all 10 ginseng ginsenoside biosynthesis genes each had 1–3 transcripts and an average of 2.2 transcripts (33% of the total number of transcripts alternatively spliced from a gene) whose expressions were significantly correlated with the ginsenoside contents in ginseng roots (Supplemental Table S4). Of the 18 cotton fiber length genes, 17 each had 1–4 transcripts and an average of 1.8 transcripts (12% of the total number of transcripts per gene) whose expressions were significantly correlated with cotton fiber length. No transcript was identified for the cotton *GhDET2* gene whose expression was correlated with cotton fiber length (Supplemental Table S5). The cotton orthologues of the Arabidopsis trichome genes, *TTG1* and *TTG2*, each had 1 or 2 transcripts (14% of the total number of transcripts per gene) whose expressions were significantly correlated with cotton fiber length (Supplemental Table S5). Together, 19 of the 20 cotton fiber length genes and cotton orthologues of the Arabidopsis trichrome genes each had 1–4 transcripts and an average of 1.8 transcripts whose expressions were significantly correlated with cotton fiber length. Similarly, all eight maize grain yield genes each had 1 or 4 transcripts and an average of 1.4 transcripts (30% of the total number of transcripts per gene) whose expressions were significantly correlated with maize grain yield (Supplemental Table S6). Of the maize orthologues of the 18 rice grain yield genes, 7 each had 1 or 2 transcripts and an average of 1.1 transcripts (13% of the total number of transcripts per gene) whose expressions were significantly correlated with maize grain yield, while no transcript was identified for the maize orthologues of the remaining 11 rice grain yield genes whose expressions were correlated with maize grain yield. The correlation analysis result was expected because some of the rice grain yield gene transcripts may not contribute to an equivalent phenotype, i.e., grain yield in maize. Together, these analysis results confirmed the functions of the published genes in the performance of the targeted traits, thus indicating that at least seven of the 18 rice grain yield genes are the candidate genes for maize grain yield.

### Relationships among the genes controlling a quantitative trait

Because the genes controlling each of the three quantitative traits were cloned by different researchers from different genetic sources and differed in nucleotide sequence and biochemical functions (Supplemental Tables S1–S3), the third question was whether they were somehow related. Since expression is essential for a gene to perform its function(s) and to shape the phenotype of a quantitative trait, we used gene transcript expressions in a tissue of different genotypes or different tissues of a single plant sampled at a developmental stage to examine whether there is any relationship among the genes controlling each of the ginseng ginsenoside biosynthesis, cotton fiber length and maize grain yield. We selected one or two transcripts per gene that were identical or the most similar to the cDNA sequences of the genes present at GenBank (Supplemental Tables S7–S9) and conducted the co-expression network analysis of the genes controlling each of these three traits. As a result, 14 transcripts were selected for the 10 ginseng ginsenoside biosynthesis genes because *β-AS*, *CAS*, *DS* and *SE2* each had two transcripts having very close identities to the cDNA sequences of the genes present at GenBank, 18 transcripts for 18 cotton fiber length genes and 2 transcripts for the cotton orthologues of the 2 Arabidopsis trichrome genes, 8 transcripts for the 8 maize grain yield genes, and 18 transcripts for the maize orthologues of the 18 rice grain yield genes. For the 10 ginseng ginsenoside biosynthesis genes, all 14 selected transcripts, with 1 or 2 transcripts per gene, were significantly correlated with the contents of ginsenosides (Supplemental Table S4). For the 18 cotton fiber length genes and the cotton orthologues of the 2 Arabidopsis trichrome genes, 15 of the 20 selected transcripts, with one transcript per gene, were significantly correlated with cotton fiber length (Supplemental Table S5), indicating that the transcript similarity to the cDNA sequences of five of the 20 genes at GenBank was inconsistent with their correlations with fiber length. For the 8 maize grain yield genes, all 8 selected transcripts, with one transcript per gene, were significantly correlated with maize grain yield, while for the 18 rice grain yield genes, 7 of the selected transcripts, with one transcript per gene, were significantly correlated with maize grain yield (Supplemental Table S6). Moreover, because any gene expressed in a cell highly likely co-expresses with one or more other genes, we randomly selected unknown gene transcripts from the corresponding transcriptomes and used as controls to check the background noise. The networks for the genes controlling each of these traits were constructed using the ginseng GWAS, cotton RIL or maize IRIL populations, respectively. Analyzed were three major components of the gene networks: the nodes representing genes, the edges representing gene-gene co-expression, and connectivity representing the robustness of a network or the number of gene nodes needed to be removed to disconnect part of a network.

**Figure 2:**
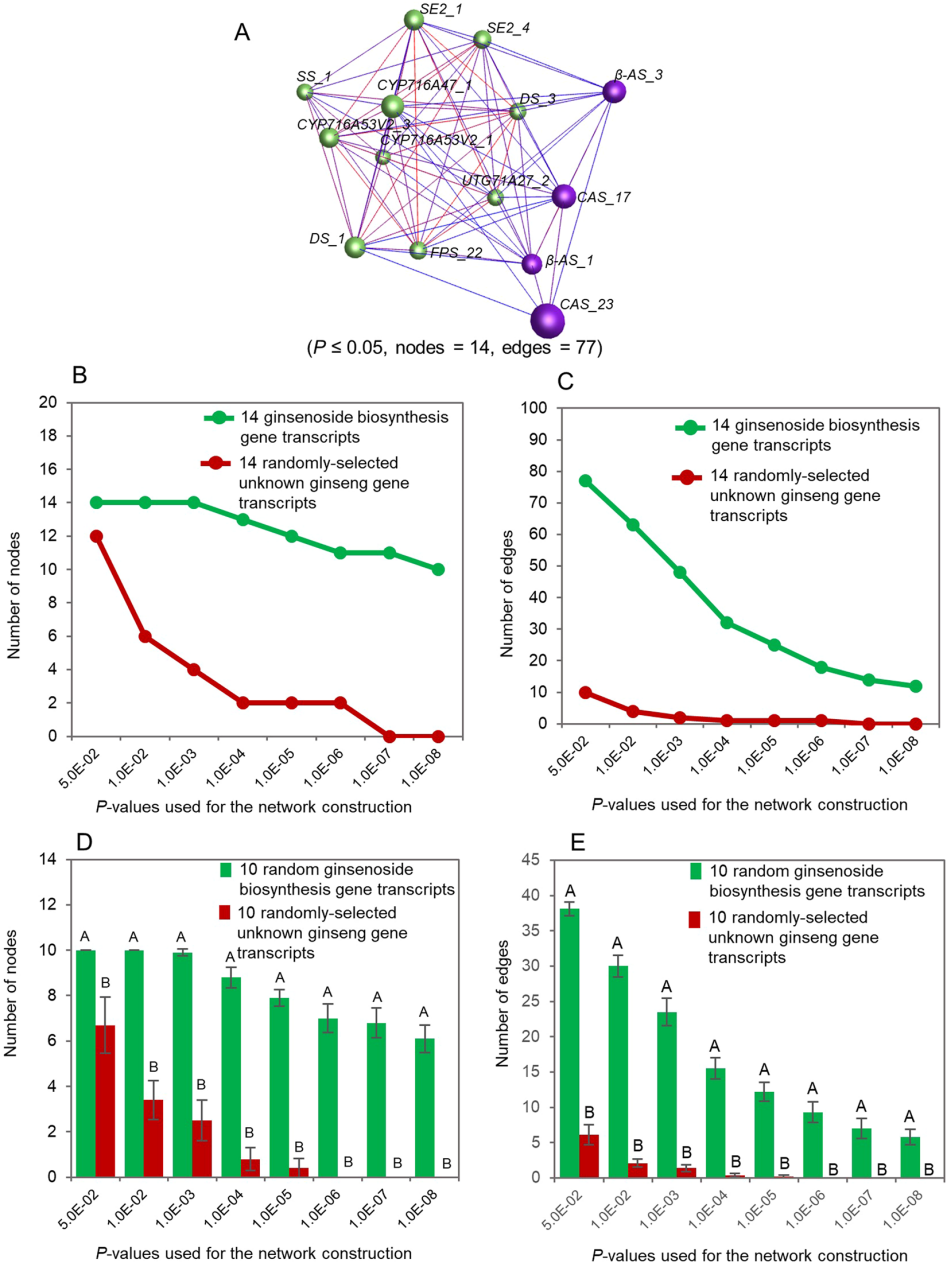
Tendency that the genes controlling ginseng ginsenoside biosynthesis form a co-expression network in the four-year-old ginseng plant roots. (**A**) The network of the 14 ginsenoside biosynthesis gene transcripts formed in the ginseng four-year-old plant roots. Nodes, gene transcripts indicated by balls; edges, gene transcript - gene transcript co-expression indicated by lines. The genes labelled in different colors indicate different Markov clusters of the network. (**B**) Tendency of the gene network formation at different *p*-values: nodes. (**C**) Tendency of the gene network formation at different *p*-values: edges. (**D**) Statistics of the gene network formation tendency: nodes. (**E**) Statistics of the gene network formation tendency: edges. The 10 ginsenoside biosynthesis gene transcripts used for the statistical analysis were randomly selected from the 14 ginsenoside biosynthesis gene transcripts used in this study, while the 10 unknown ginseng gene transcripts were randomly selected from a ginseng four-old-year plant database^16^ using bootstrap sampling with 100 replications. Error bar, standard deviation for the 100 replications; different capital letters, significant at *P* ≤ 0.01 determined by Student’s *t*-test.

**Figure 3:**
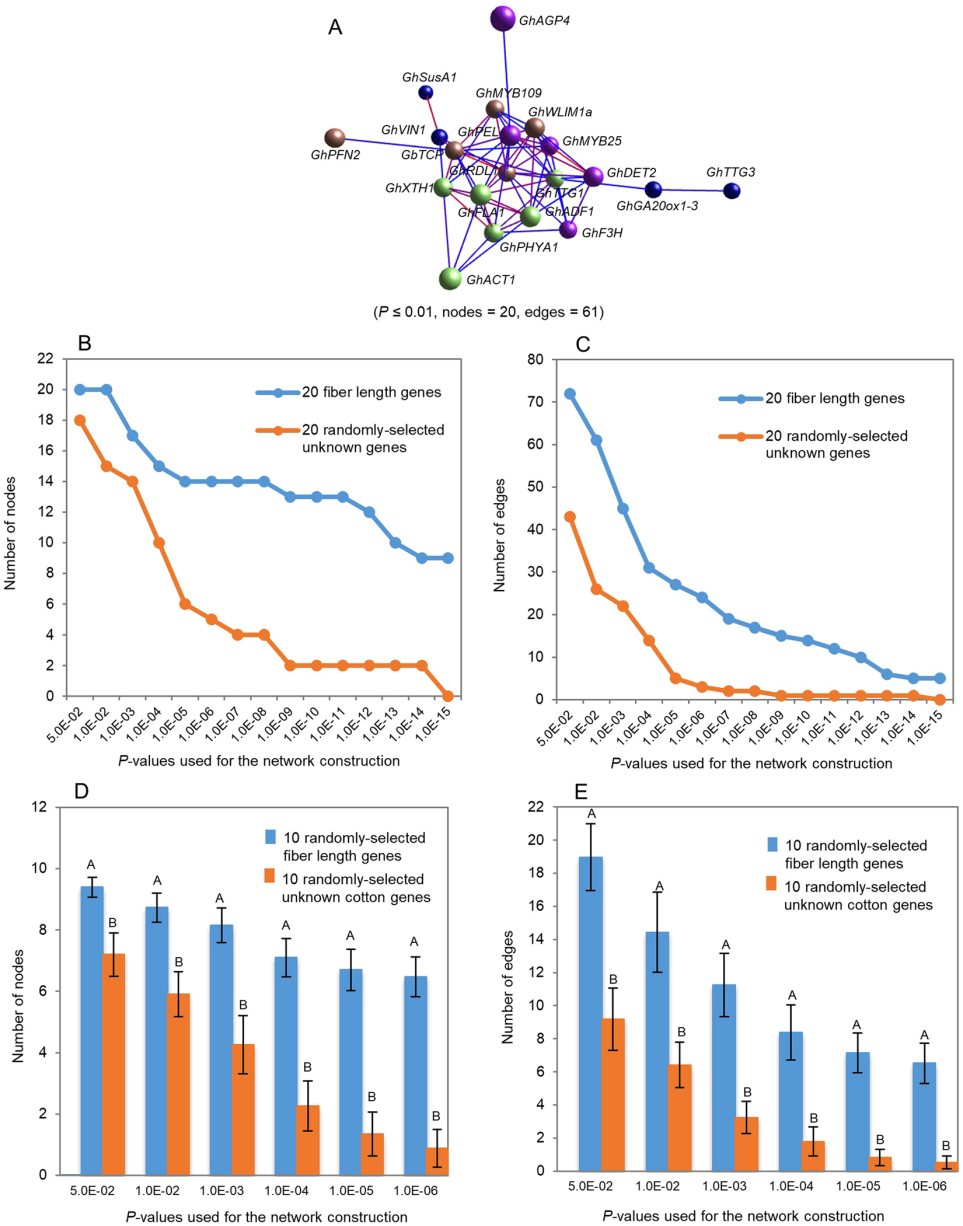
Tendency that the genes controlling cotton fiber length form a co-expression network in developing fibers on the 10th day after flowering (10-dpa fiber). Each gene is presented by one of its transcripts. (**A**) The network of the 20 cotton fiber length gene transcripts formed in 10-dpa fibers. Nodes, gene transcripts indicated by balls; edges, gene transcript-gene transcript co-expressions indicated by lines. The genes labelled in different colors indicate different Markov clusters of the network. (**B**) Tendency of the gene network formation at different *p*-values: nodes. (**C**) Tendency of the gene network formation at different *p*-values: edges. (**D**) Statistics of the gene network formation tendency: nodes. (**E**) Statistics of the gene network formation tendency: edges. The 10 fiber length gene transcripts used for the statistical analysis were randomly selected from the 20 fiber gene transcripts used in this study while the 10 unknown cotton gene transcripts were randomly selected from a cotton 10-dpa fiber database^17^ using bootstrap sampling with 100 replications. Error bar, standard deviation for the 100 replications; different capital letters, significant at *P* ≤ 0.01 determined by Student’s *t*-test.

**Figure 4:**
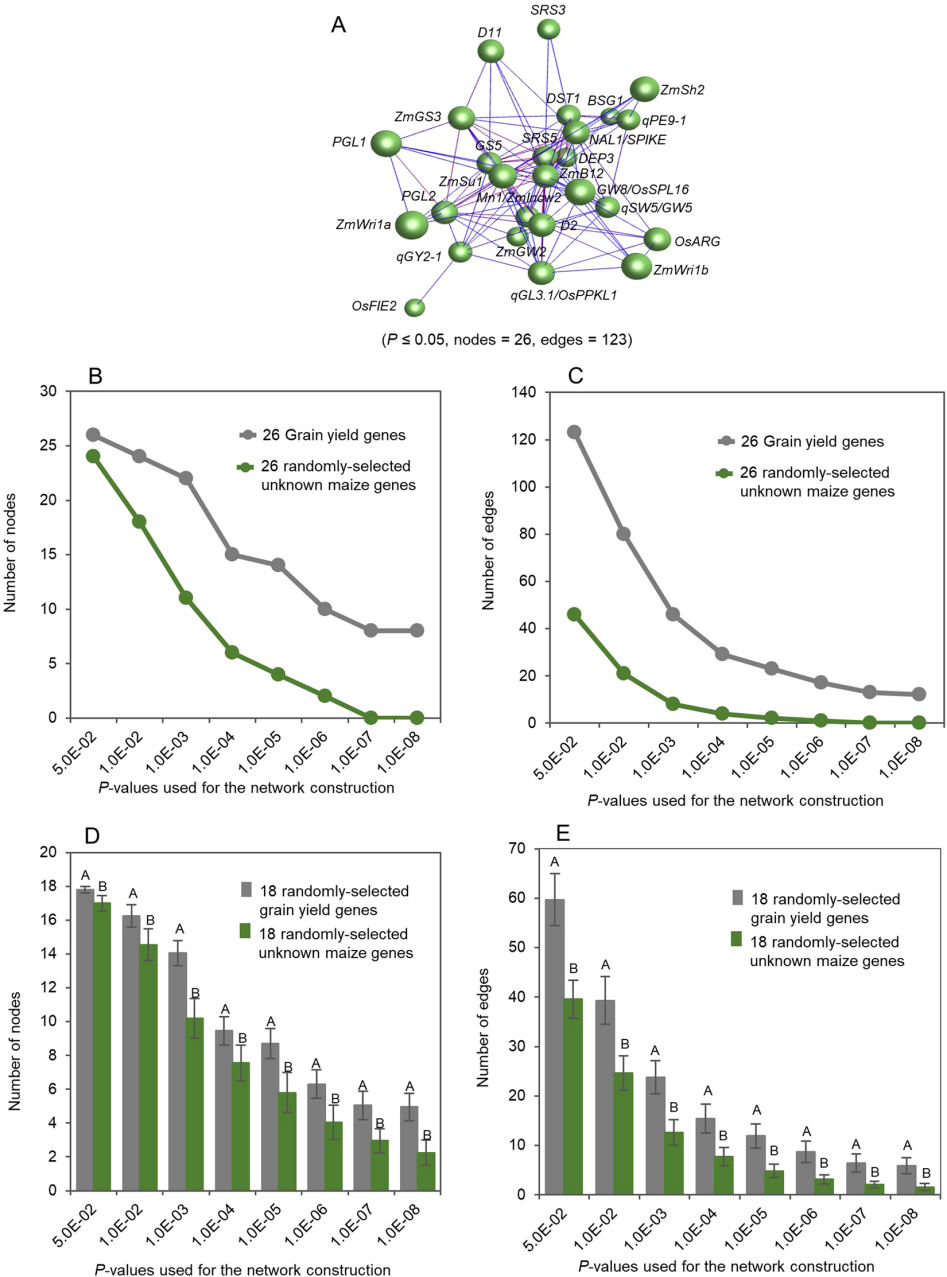
Tendency that the genes controlling maize grain yield form a single co-expression network in 13-leaf top ear shoots. Each gene is presented by one of its transcripts. (**A**) The network of the 8 maize and 18 rice grain yield gene transcripts formed in developing top ear shoots of maize at the 13-leaf stage. Nodes, gene transcripts indicated by balls; edges, gene transcript-gene transcript co-expressions indicated by lines. The network consists of only one Markov cluster. (**B**) Tendency of the gene network formation at different *p*-values: nodes. (**C**) Tendency of the gene network formation at different *p*-values: edges. (**D**) Statistics of the gene network formation tendency: nodes. (**E**) Statistics of the gene network formation tendency: edges. The 18 grain yield genes used for the statistical analysis were randomly selected from the 26 grain yield genes used in this study while the 18 unknown maize genes were randomly selected from a 13-leaf top ear shoot database^17^ using bootstrap sampling with 100 replications. Error bar, standard deviation for the 100 replications; different capital letters, significant at *P* ≤ 0.01 determined by Student’s *t*-test.

We first tested the tendency that the gene transcripts controlling a quantitative trait formed a co-expression network in a tissue sampled at a developmental stage in a bi-parental population or a GWAS panel. For ginseng ginsenoside biosynthesis, the 14 selected transcripts of the 10 genes controlling this biological process formed a single co-expression network that consisted of two clusters, when a cutoff of *P* ≤ 0.05 was applied (Fig. 2A). The network consisted of all 14 ginsenoside biosynthesis gene transcripts selected for the network construction and 77 gene transcript-gene transcript co-expression edges. In comparison, the numbers of both nodes and edges constituting the networks of the ginsenoside biosynthesis gene transcripts were higher than those of the randomly selected unknown ginseng gene transcripts (*P* ≤ 0.01), no matter what *P*-values, from *P* = 5.0E-02 to *P* = 1.0E-08, were used for network construction (Fig. 2B-E). For cotton fiber length, the selected transcripts of the 20 fiber length or trichome genes also formed a single co-expression network consisting of three clusters, when a cutoff of *P* ≤ 0.01 was applied (Fig. 3A). The network consisted of all the 20 gene transcripts used for the network construction and 61 gene transcript-gene transcript co-expression edges. The numbers of both nodes and edges of the network were higher than those of the randomly-selected unknown cotton gene transcripts (*P* ≤ 0.01), no matter what *P*-values, from *P* = 5.0E-02 to *P* = 1.0E-08, were used for network construction (Fig. 3B-E). For maize grain yield, similar results were achieved, even though 18 of the 26 genes used for the analysis were known to control grain yield in rice. The functions of 14 of the 18 rice grain yield genes in maize were unknown and four were shown to also contribute to grain yield in maize^12–15^. Again, all the 26 grain yield genes used for the network construction formed a single co-expression network consisting of a single cluster, when a cutoff of *P* ≤ 0.05 was applied (Fig. 4A). The network consisted of all the 26 grain yield gene transcripts used for the network construction and 123 gene transcript-gene transcript co-expression edges. The numbers of both nodes and edges of the network were higher than those of the randomly-selected unknown maize genes (*P* ≤ 0.01), no matter what *P*-values, from *P* = 5.0E-02 to *P* = 1.0E-08, were used for network construction (Fig. 4B-E). These results consistently indicated that the genes controlling a quantitative trait were much more likely to form a co-expression network than the randomly-selected unknown genes, no matter which trait was tested, which species the trait was from and where the plants were grown.

**Figure 5:**
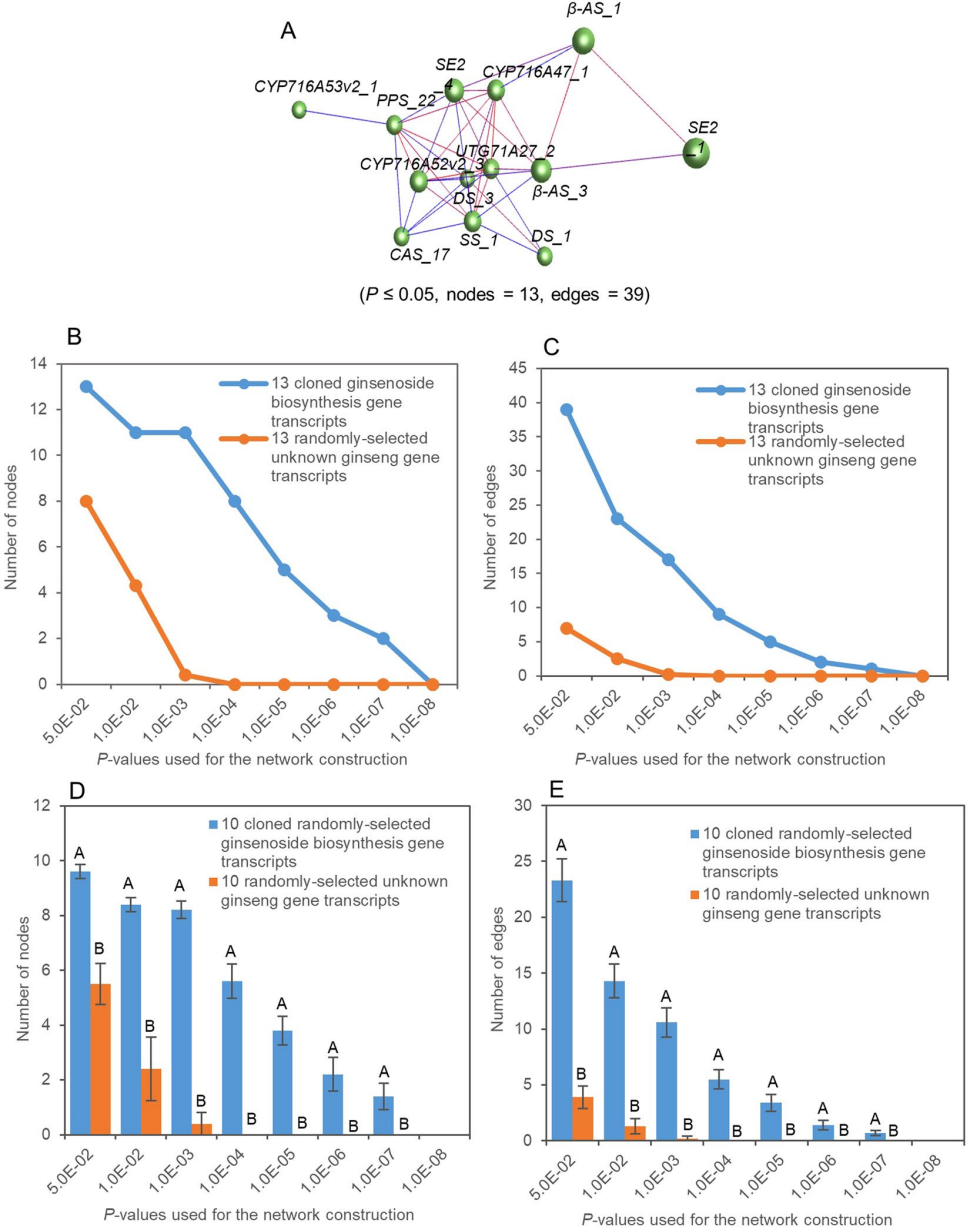
Tendency that the genes controlling ginseng ginsenoside biosynthesis form a single co-expression network in a four-year-old ginseng plant at the fruiting stage. (**A**) The network of the 13 ginsenoside biosynthesis gene transcripts formed in 14 tissues of a four-year-old ginseng plant. Nodes, gene transcripts indicated by balls; edges, gene transcript - gene transcript co-expressions indicated by lines. One of the 14 ginsenoside biosynthesis gene transcripts, *CAS_23*, was excluded from the analysis because it did not express in the 14 tissues of the plant. The network consists of only one Markov cluster. (**B**) Tendency of the gene network formation at different *p*-values: nodes. (**C**) Tendency of the gene network formation at different *p*-values: edges. (**D**) Statistics of the gene network formation tendency: nodes. (**E**) Statistics of the gene network formation tendency: edges. The 10 ginsenoside biosynthesis gene transcripts used for the statistical analysis were randomly selected from the 13 ginsenoside biosynthesis gene transcripts used in this study, while the 10 unknown ginseng gene transcripts were randomly selected from a ginseng four-old-year plant database^16^ using bootstrap sampling with 100 replications. Error bar, standard deviation for the 100 replications; different capital letters, significant at *P* ≤ 0.01determined by Student’s *t*-test.

Then, we further confirmed the significantly higher tendency that the genes controlling a quantitative trait formed a co-expression network than the randomly selected unknown genes using 14 tissues of a four-year-old ginseng plant sampled at the fruiting stage. Since only 13 of the 14 selected transcripts of the 10 ginsenoside biosynthesis genes expressed in two or more of the tissues of the plant that are necessary for proper calculation of correlation coefficient for network construction, the 13 transcripts were used for this experiment. When a cutoff of *P* ≤ 0.05 was applied, all the 13 transcripts again formed a single co-expression network (Fig. 5A). The network consisted of all 13 ginsenoside biosynthesis gene transcripts used for the network construction and 39 gene transcript-gene transcript co-expression edges. In comparison, the numbers of both nodes and edges constituting the networks of the ginsenoside biosynthesis gene transcripts were higher than those of the randomly selected unknown ginseng gene transcripts (*P* ≤ 0.01), no matter what *P*-values, from *P* = 5.0E-02 to *P* = 1.0E-08, were used for network construction (Fig. 5B-E).

**Figure 6:**
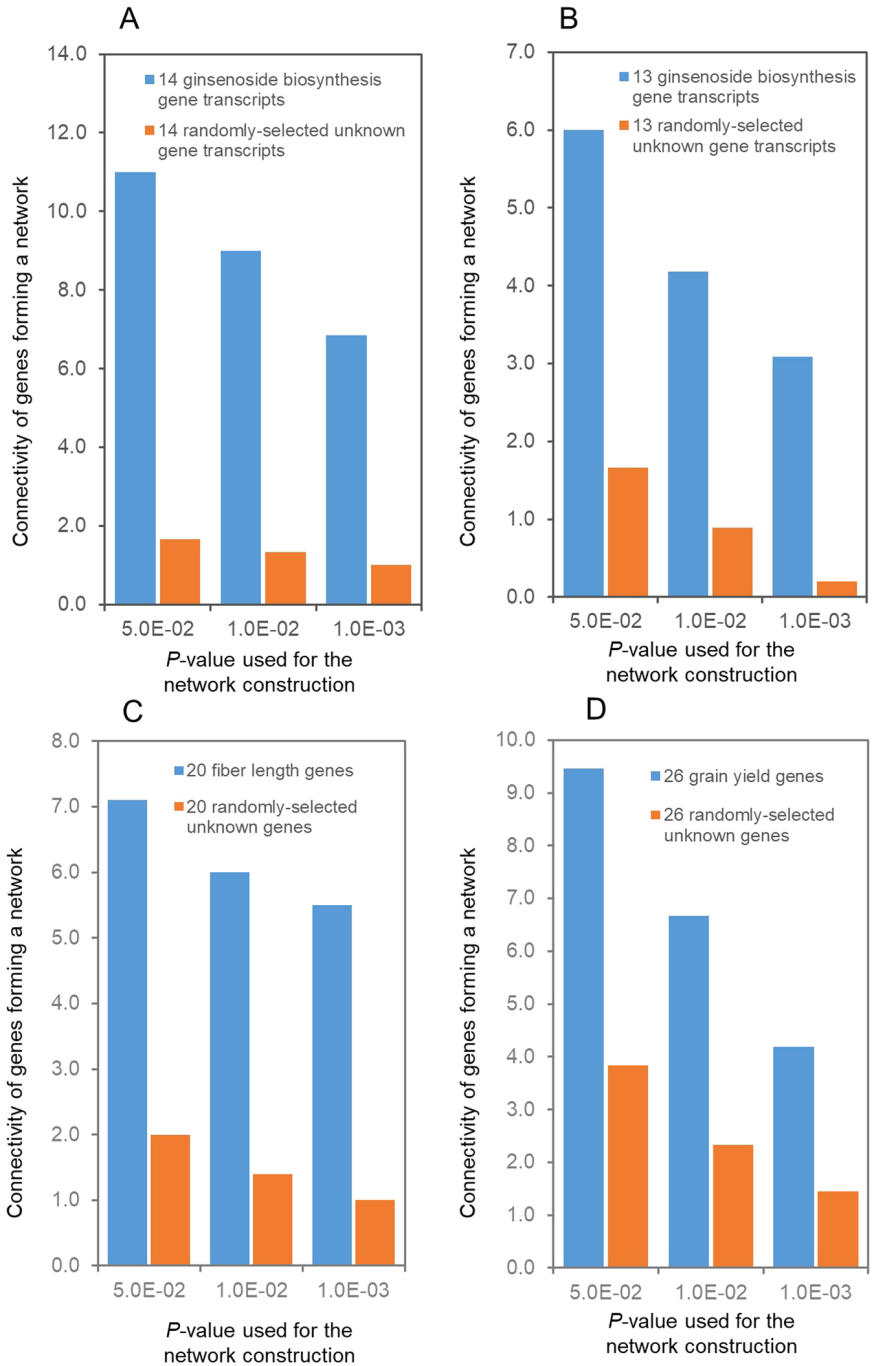
Connectivity of the genes controlling a quantitative trait forming a co-expression network. The network connectivity is a measure of network robustness, presented by the number of gene nodes needed to be removed to disconnect part of a network. (**A**) The network of ginsenoside biosynthesis genes in four-year-old plant roots of different ginseng cultivars. (**B**) The network of ginsenoside biosynthesis genes in 14 tissues of a four-year-old ginseng plant. One of the 14 ginsenoside biosynthesis gene transcripts, *CAS_23*, was excluded from this analysis because it did not express in the 14 tissues of the plant. (**C**) The network of cotton fiber length genes in developing fibers of different cotton lines sampled on the 10th day post-anthesis (10-dpa). (**D**) The network of maize grain yield genes in 13-leaf top ear shoots.

Furthermore, we examined the connectivity of the co-expression networks of the genes controlling ginseng ginsenoside biosynthesis, cotton fiber length, and maize grain yield against those of the gene transcripts randomly selected from corresponding transcriptome databases at three *p*-values for network construction, respectively. In the four-year-old plant roots of the 42 ginseng lines, the connectivity of the network formation of the 14 ginsenoside biosynthesis gene transcripts was 6.6-, 6.8-, and 6.9-fold at a *p*-value of 0.05, 0.01, and 0.001, respectively, as those of the 14 randomly-selected unknown ginseng gene transcripts (Fig. 6A). In the 14 tissues of the four-year-old ginseng plant, the connectivity of the network formation of the 13 ginsenoside biosynthesis gene transcripts was 3.6-, 4.7-, and 15.5-fold at a *p*-value of 0.05, 0.01, and 0.001, respectively, as those of the 13 randomly-selected unknown ginseng gene transcripts (Fig. 6B). Similarly, the connectivity of the 18 cotton fiber length gene transcripts and those of the cotton orthologues of the 2 Arabidopsis trichrome gene transcripts forming co-expression networks in cotton 10-dpa developing fibers were 3.6-, 4.3-, and 5.5-fold at a *p*-value of 0.05, 0.01, and 0.001, respectively, as those of the 20 randomly-selected unknown cotton gene transcripts (Fig. 6C). The connectivity of the 8 maize grain gene transcripts and those of the maize orthologues of the 18 rice grain yield gene transcripts forming co-expression networks in maize developing top ear shoots were 2.5-, 2.9-, and 2.9-fold at a *p*-value of 0.05, 0.01, and 0.001, respectively, as those of the 26 randomly-selected unknown maize gene transcripts (Fig. 6D).

### The co-expression network of the genes controlling a quantitative trait and its phenotype

**Figure 7:**
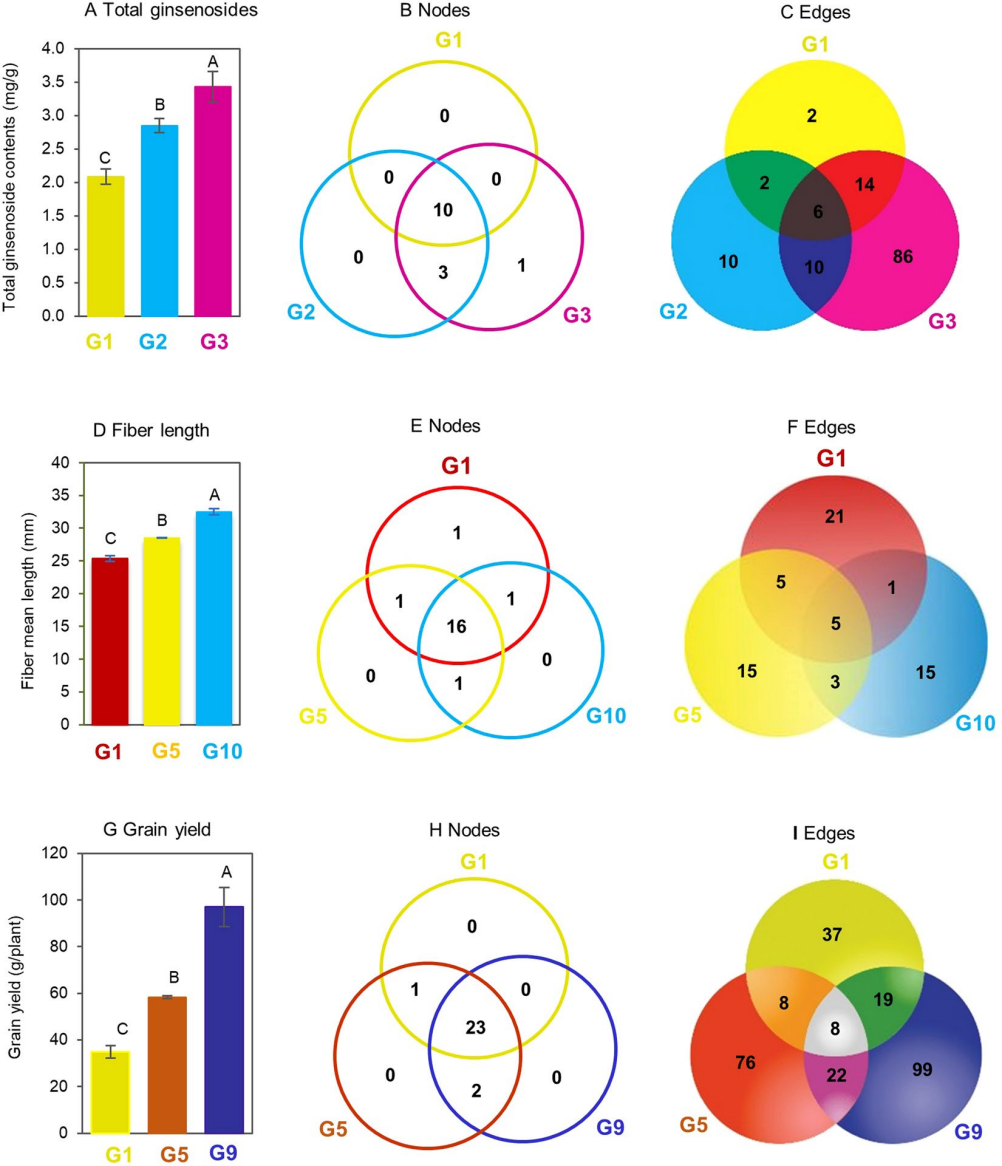
Relationship between variation in the network of the genes controlling a quantitative trait and phenotype variation in the trait that they control. The networks were constructed at a cutoff of *P* ≤ 0.01. (**A** to **C)** Ginseng. Fourteen lines that had similar ginsenoside contents were included in each of the groups, G1, G2, and G3, for the analysis. (**D** to **F**) Cotton. twenty RILs that had similar fiber lengths were included in each of the groups, G1, G5, and G10, for the analysis. (**G** to **I**) Maize. Ten IRILs that had similar grain yields were included in each of the groups, G1, G5, and G9, for the analysis. Error bar, standard deviation for the lines of each group; different capital letters, significant at *P* ≤ 0.01 determined by ANOVA, followed by LSD.

Since the genes controlling a quantitative trait were coordinated in functional activity, the fourth question was whether this relationship is consistent across genotypes and if it is not, whether variation in the gene network is somehow related with the performance of the targeted trait that the genes control. We grouped the 42 ginseng GWAS lines into three groups, with each group consisting of 14 lines, according to their ginsenoside contents in four-year-old plant roots. Group 1 (G1) had the lowest ginsenoside content, G2 had the middle ginsenoside content, and G3 had the highest ginsenoside content. The ginsenoside contents of these three groups differed by 26–61% (*P* ≤ 0.01 determined by ANOVA followed by LSD) (Fig. 7A). We grouped the 198 cotton RILs and their two parents into ten groups according to their fiber lengths, from G1 to G10 having an ascending fiber length and with each group consisting of 20 lines. G1, G5, and G10 were used for this experiment and their fiber lengths differed by 9–27% (*P* ≤ 0.01) (Fig. 7D). We grouped 88 of the 89 maize IRILs and their two parents into nine groups according to their grain yields, from G1 to G9 having an ascending grain yield and with each group consisting of 10 lines. G1, G5, and G9 were used for this experiment and their grain yields differed by 63–172% (*P* ≤ 0.01) (Fig. 7G). We separately constructed the networks of the genes controlling the traits for each group and compared the networks of the genes controlling each trait among the groups, specifically in number of nodes, number of edges and structure of the network including which of the genes were in the network and with which genes a gene co-expressed (Fig. 7B,C,E,F,H,I). The network of the genes controlling each trait was found to substantially vary in the number of nodes, number of edges and network structure, in association with the variation in phenotype of the targeted trait that they control. This result suggested that the variation in the network of genes controlling a quantitative trait was associated with the performance of the targeted trait. The nodes of the gene networks were much more stable across genotypes or groups differing in phenotype than their co-expression edges. The nodes had a consistence of 71% across genotypes with significantly different phenotypes for ginseng ginsenoside biosynthesis, 80% for cotton fiber length, and 88% for maize grain yield, whereas the edges had a consistency of only 11% across genotypes with significantly different phenotypes for ginseng ginsenoside biosynthesis, 19% for cotton fiber length, and 7% for maize grain yield. Therefore, the phenotype of a quantitative trait might be determined not only by the node members of its gene network, but also by its gene collaboration or interaction in functional activity.

## Discussion

The previous cloning of multiple genes controlling each of ginseng ginsenoside biosynthesis, cotton fiber length, and maize grain yield has confirmed classical quantitative genetics that a trait showing quantitative inheritance, i.e., a quantitative trait, is controlled by numerous genes. This study has further made several new insights into the molecular basis of quantitative genetics. First, this study reveals that a vast majority of the genes controlling a quantitative trait (cotton fiber length and maize grain) or a polygenic biological process (ginseng ginsenoside biosynthesis) are alternatively spliced into multiple transcripts, thus likely having multiple functions or being pleiotropic, because each transcript may code a different protein having a different biological function. Second, the genes controlling a quantitative trait express correlatively, forming a multiple-time stronger co-expression network than other genes in an organ, regardless of their sequence identity, annotations, and/or GO categorization. Third, the network of the genes controlling a quantitative trait varies substantially among genotypes differing in phenotype of the trait that they control, suggesting the roles of gene network in trait performance. These three conclusions are held for all three traits studied using all three species, no matter where the plants were grown, suggesting that they is stable across traits, across species and across environments. Furthermore, the molecular characteristics of the genes controlling maize grain yield and cotton fiber length have been further validated by analysis of the 1,501 maize grain yield genes^21^ and the 474 cotton fiber length genes^22^ that we recently cloned genome-wide (in preparation). Therefore, we conclude that a quantitative trait is a consequence of collaboration among its controlling genes, not only the gene members of its gene network, but also the gene relationships in the network, such as whether they collaborate or not in activity and with which gene(s) a gene of the network co-expresses. This finding suggests that it is essential to decipher the molecular mechanism underlying a quantitative trait, and to develop new or advanced methods efficient for the trait manipulation, such as gene editing, by genome-wide analysis of the genes controlling that trait.

Genes are the keys to developing new or advanced technologies for enhanced breeding in plants and livestock, and for enhanced medicine in humans. Nevertheless, only a limited number of genes controlling each quantitative trait has been so far cloned. It is apparent that many more genes remain to be identified in order to comprehensively decipher their underlying molecular mechanisms and to develop new or advanced technologies for their efficient manipulation for enhanced breeding and enhanced medicine. The significant correlation between transcript expression of the genes and phenotypic variation of the trait that they control in a population and their multiple-fold higher tendency in formation of a co-expression network revealed in this study provide useful information for genome-wide identification of the genes controlling a quantitative trait, not only the genes controlling quantitative traits in these three species, but also those controlling quantitative traits in other species using a cloned gene controlling the trait as a start point. In this study, we have, using this information, identified seven candidate genes controlling grain yield in maize (Fig. 4; Supplemental Table S6).

Finally, this study reveals that most of the genes controlling a quantitative trait are subjected to RNA alternative splicing and spliced into multiple transcripts. It is apparent that only one or a limited number, but not all, of the transcripts alternatively spliced from a multiple transcript gene is involved in phenotype development of the trait. These results raise an extremely significant question about gene research and characterization: whether gene or its individual transcripts are used as basic functional units for gene research, characterization and application, such as gene annotation, pathway construction, functional categorization, gene editing, and genetic engineering. This finding supports for our previous proposal that individual gene transcripts should be used for these purposes of gene research^17^.

## Supplementary information

Supplementary Figure S1

Supplementary Figure S2

Supplementary Figure S3

Supplementary Figure S4

Supplementary Information

Supplementary Table S1

Supplementary Table S2

Supplementary Table S3

Supplementary Table S4

Supplementary Table S5

Supplementary Table S6

Supplementary Table S7

Supplementary Table S8

Supplementary Table S9
