# Supplementary figures and images for: Analysis of the genes controlling three quantitative traits in three diverse plant species reveals the molecular basis of quantitative traits
Source: Sci Rep. 2020 Jun 22;10:10074. doi: 10.1038/s41598-020-66271-8 (PMC7308372; doi:10.1038/s41598-020-66271-8)

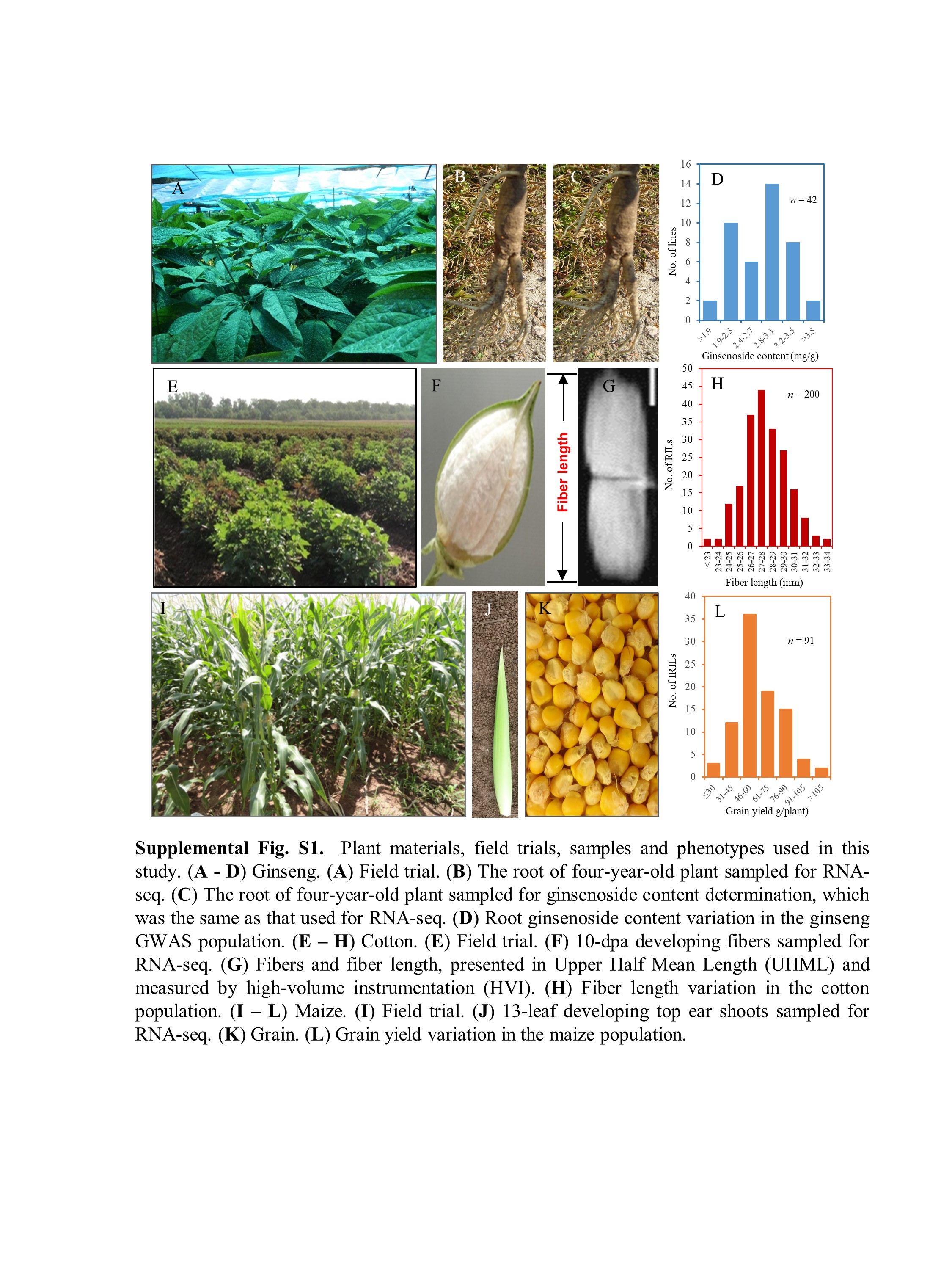

Supplement: Supplementary file 1 — Supplementary Figure S1 [file 41598_2020_66271_MOESM1_ESM.jpg]

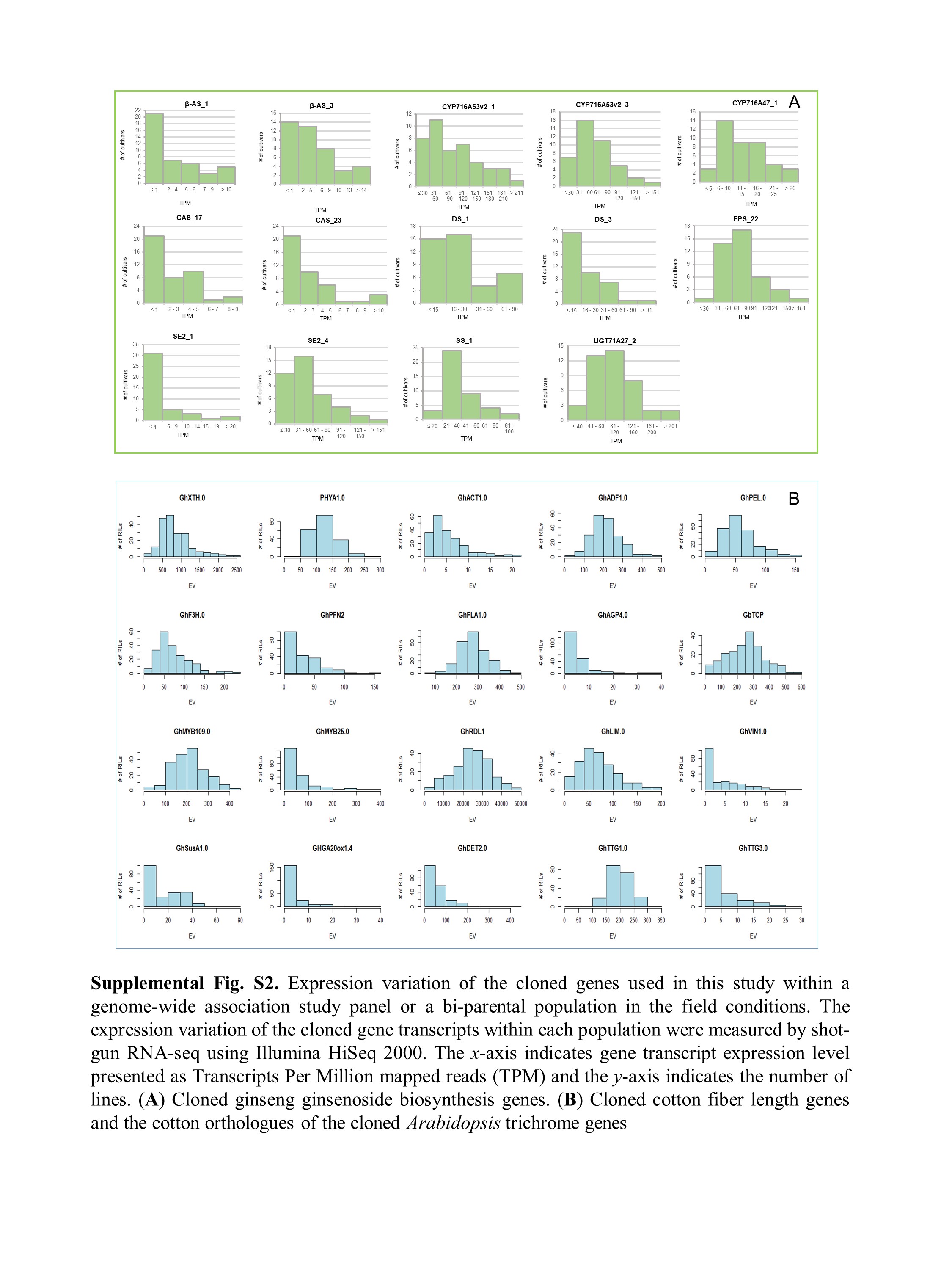

Supplement: Supplementary file 2 — Supplementary Figure S2 [file 41598_2020_66271_MOESM2_ESM.jpg]

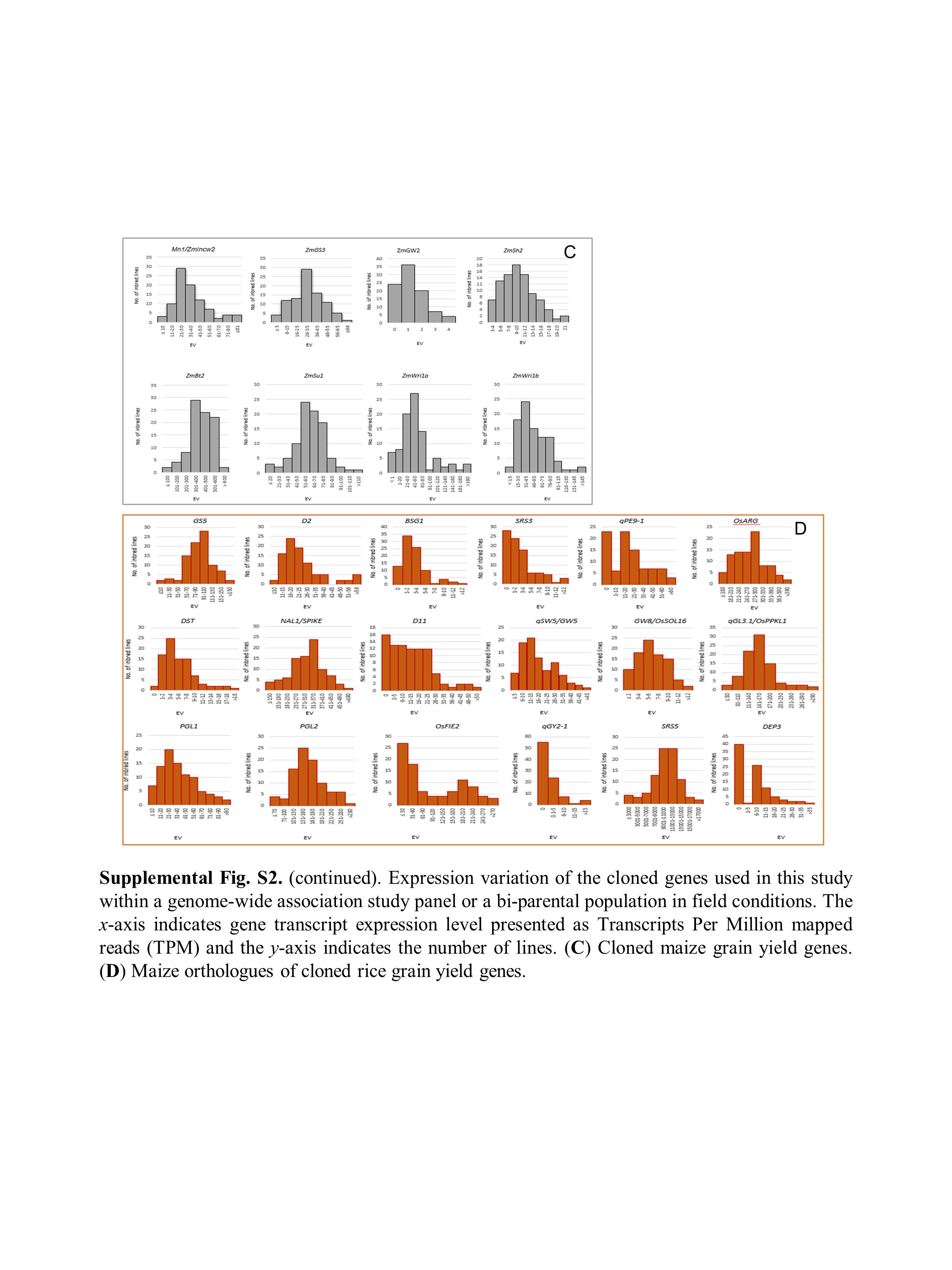

Supplement: Supplementary file 3 — Supplementary Figure S3 [file 41598_2020_66271_MOESM3_ESM.jpg]

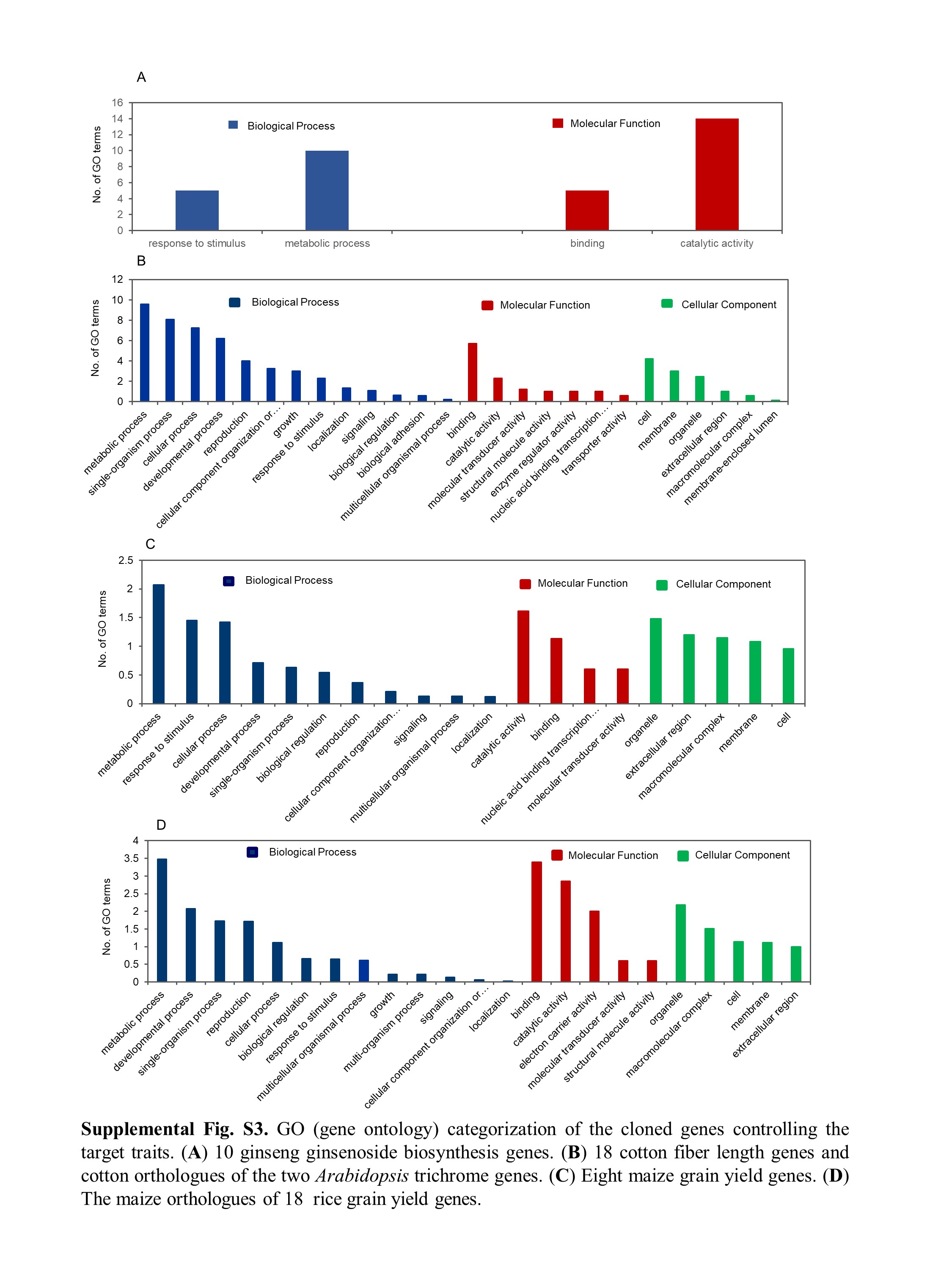

Supplement: Supplementary file 4 — Supplementary Figure S4 [file 41598_2020_66271_MOESM4_ESM.jpg]
