# Supplementary material for: Analysis of the genes controlling three quantitative traits in three diverse plant species reveals the molecular basis of quantitative traits
Source: Sci Rep. 2020 Jun 22;10:10074. doi: 10.1038/s41598-020-66271-8 (PMC7308372; doi:10.1038/s41598-020-66271-8)
Supplement: Supplementary file 5 — Supplementary Information [file 41598_2020_66271_MOESM5_ESM.docx]

Supplemental Data for the Article:

**Analysis of the genes controlling three quantitative traits in three diverse plant species reveals the molecular basis of quantitative traits**

**Meiping Zhang^1,2^, Yun-Hua Liu^2^, Wenwei Xu^3^, C. Wayne Smith^2^, Seth C. Murray^2^ & Hong-Bin Zhang^2,^***

^1^College of Life Science, Jilin Agricultural University, Changchun, Jilin 130118, China. ^2^Department of Soil and Crop Sciences, Texas A&M University, College Station, Texas 77843, United States of America.

^3^Texas A&M AgriLife Research, Lubbock, TX 79403, United States of America.

Meiping Zhang and Yun-Hua Liu contributed equally to this work.

*Corresponding author (email: [hbz7049@tamu.edu](mailto:hbz7049@tamu.edu))

**Supplemental Data:**

Supplemental Figures 1 to 3

Supplemental Tables 1 to 9
